# Annotations

**Published:** 1900-11-24

**Authors:** 


### Annotations.

### Lord Rosebery's Rectorial Address.

Many who read the admirable address given by the Earl of Rosebery on his installation as Lord Rector of the Glasgow University must have wondered and regretted that one gifted with such lofty ideals should have allowed himself to dally so long outside the stream of practical politics. Nothing could have been more vigorous or more telling than what he said as to the competition in every field to which Englishmen in the twentieth century will of a certainty be exposed, and of the necesity of our maintaining our characteristics as an Imperial race if we would avoid being pushed into the background. And what is an Imperial race? It is, as Lord Rosebery says, "a race vigorous, and industrious, and intrepid." But when he asks, "Are we raising such a race?" he points to one of the weak spots in England's present position, and one which it is to be feared we are but making clumsy efforts to eradicate. In the rural districts, he says, the children may be matched against any children in the world, "but in the great cities—in the rookeries and slums which still survive—an Imperial race cannot be reared. You can scarcely produce anything in those foul nests of crime and disease but a progeny doomed from its birth to misery and ignominy. That is a rift in the cornerstone of your commonwealth." Turning, then, to those who were embarking on the profession of medicine, he said with great truth, and with a grasp of the importance of sanitation and of the health of the individual by which the statesman should always be actuated, "Remember that where you promote health and arrest disease, where you convert an unhealthy citizen into a healthy one, where you exercise your authority to promote sanitary conditions and suppress those which are the reverse, you, in doing your duty, are also working for the Empire." Nothing could be higher than the standard set up by Lord Rosebery, nothing could be more thorough than the way in which he laid bare the risks and dangers which are now being run by the English people of losing those characteristics by which they have become an Imperial race. He showed that reform is not merely a matter of institutions, but of manners, of habits, and of men; that as to the breeding of the race itself, notwithstanding all the comfortable doctrines of the optimists, "the survival of the fittest is an absolute truth in the conditions of the modern world"; and that to attain this fitness, while the Universities trained the national mind, the national body must not be neglected. He hoped, therefore, that the medical officer of health might be armed with sufficient power—a power not derived merely from authority, but supported by public opinion.

### Camp Sanitation.

Two letters have recently appeared in the *Times* on the subject of camp sanitation which strongly suggest that sanitary matters have not received that attention at the hands of the military authorities which they deserve. A regimental officer with the South African Field Force points out that in standing camps the water difficulty is to a large extent a matter of lack of careful supervision of the arrangements for filtration. All units are now provided with first-class filters, which, if properly attended to, are, he says, an inestimable boon. In numerous cases, however, these filters have been rendered useless by unfair treatment, becoming rapidly clogged up by the mud with which so much of the water in South Africa is highly charged; and he points out how easily this difficulty can be overcome by a preliminary straining. The fact is the provision of pure water has to be taken seriously, and when we are gravely told that in standing camps in which filters are at work it is "impossible" to prevent our soldiers, so well disciplined in every other respect, from helping themselves to water which is officially regarded as suspect, we cannot doubt that the task of protecting the water supply has hardly been undertaken with that sense of its importance which the subject demands. The necessity of placing the water under the guardianship, not of a mere sentry, but of an officer of some position and authority was one of the points insisted on when the subject of camp sanitation was discussed at the International Medical Congress in Paris.

The other point raised is as to the latrines, it being stated that the sanitary arrangements for our soldiers when in camp round about Aldershot are given over to contractors, who provide receptacles and remove them as required—which is all very nice and saves the men a certain amount of disagreeable work, with, however, this obvious disadvantage, that they go through their training without practical experience in the essential art of living healthily in camp.

### Stands Glasgow Where it Did?

The claim of Glasgow to be regarded as a model municipality has been so widely advertised that the city authorities cannot expect to escape the wondering censure of their neighbours at the possibility of such a case as this:—Henry Smith was attacked with small-pox, and was removed to the City Isolation Hospital at Belvidere. His house was then disinfected, and his housekeeper and his two children were revaccinated. One of the sanitary inspectors then gave them a line of admission to the South York Street reception house. Having given the woman and children this line, the inspector locked up Smith's house and took possession of the key. But the "suspects" were refused admission to the reception house, and returned home. They found the door shut, and none of their neighbours would give them shelter. Therefore they lay down to sleep on the landing outside their home. On the complaint of one of the neighbours a policeman aroused them, and took them to the local police office. From there they were sent to the district police office, and then to another reception house. No conveyance was used, the woman and two children of five or six had to walk all the way from one place to another, when they were already worn out with fatigue and want of sleep, and might, for aught the authorities knew, have been suffering from the first stages of a dangerous disease. At four o'clock in the morning they were refused admission to the second reception house. The constable who had accompanied them to it was taking them back to the police office, but the woman refused to walk further, and sat down on a seat in Cathedral Square. There, at five in the morning, the policeman left them, and reported the case at the office; but nothing is known of their movements until between two and three o'clock in the afternoon, when they were seen by the officer who had revaccinated them. He then secured for them admission to the South York Street reception house, where they had been refused admission the previous evening. The case was inquired into by a sub-committee of the Corporation of Glasgow, who have granted compensation to the sufferers. In a note the committee adds:—"Viewed personally, this is a sad case; municipally it is a regrettable one, and officially it is a discreditable one." And most people will agree with the committee.

